# Bioactive Polymeric Materials for the Advancement of Regenerative Medicine

**DOI:** 10.3390/jfb12010014

**Published:** 2021-02-20

**Authors:** Anthony Iovene, Yuwen Zhao, Shue Wang, Kagya Amoako

**Affiliations:** 1 Biomedical Engineering Graduate Program, Biomedical Engineering Faculty, Tagliatela College of Engineering, University of New Haven, 300 Boston Post Rd., West Haven, CT 06516, USA; aiove1@unh.newhaven.edu (A.I.); yzhao6@unh.newhaven.edu (Y.Z.); 2 Department of Mechanical, Industrial and Biomedical Engineering, Biomedical Engineering Faculty, Tagliatela College of Engineering, University of New Haven, 300 Boston Post Rd., West Haven, CT 06516, USA; 3 Biomaterials and Medical Device Innovation Laboratory, Biomedical Engineering Faculty, Tagliatela College of Engineering, University of New Haven, 300 Boston Post Rd., West Haven, CT 06516, USA

**Keywords:** biopolymers, polymer modification, bioactivity, regenerative medicine, tissue regeneration

## Abstract

Biopolymers are widely accepted natural materials in regenerative medicine, and further development of their bioactivities and discoveries on their composition/function relationships could greatly advance the field. However, a concise insight on commonly investigated biopolymers, their current applications and outlook of their modifications for multibioactivity are scarce. This review bridges this gap for professionals and especially freshmen in the field who are also interested in modification methods not yet in commercial use. A series of polymeric materials in research and development uses are presented as well as challenges that limit their efficacy in tissue regeneration are discussed. Finally, their roles in the regeneration of select tissues including the skin, bone, cartilage, and tendon are highlighted along with modifiable biopolymer moieties for different bioactivities.

## 1. Introduction

Regenerative medicine is a rapidly growing multidisciplinary field that applies biological, chemical and engineering principles to promote tissue regeneration. The goal of regenerative medicine is the repair, restoration or regeneration of lost or injured tissues using biomaterials, living cells and different signaling factors (i.e., growth factors) [1]. It has been reported in recent decades that regenerative medicine has significantly developed in terms of tissue repair and restoration including the cartilage [2,3,4], skin [5,6], bone [7,8,9,10,11,12] and blood vessels [13,14,15] using various biopolymeric materials. By responding to stimuli from the surrounding environment, living cells interact with biopolymeric materials to enhance tissue regeneration or restoration. As the interaction between materials and the cells are important, appropriate signals generated by biopolymeric materials to guide cells towards desirable behaviors as required by different types of tissue restoration or regeneration inside the body is a leading focus in regenerative medicine. 

The basic types of biomaterials that have been broadly used in regenerative medicine can be classified as natural and synthetic polymeric biomaterials. Both natural and synthetic biomaterials alone or in combination possess significant importance in regenerative medicine due to their good biocompatibility, biodegradability, and bioactivity. Specifically, to regenerate or restore different types of tissues, the combination of different biopolymers must acquire appropriate mechanical, physical and biological properties and this can be achieved due to their chemical and mechanical tunability. Furthermore, the important factors in tissue regeneration to meet the different tissues’ complex functionalities are the scaffold architecture, biodegradability, physical stability, and vascularization. For example, scaffolds made of polymeric biomaterials should be able to enhance cell biomaterial interactions to achieve controllable cellular adhesion, proliferation, differentiation, and material degradation. Among all these factors, tunable bioactivity of either natural, synthetic, or a combination of both polymer types plays an important role in tissue restoration and regeneration. 

Natural polymeric materials have been greatly investigated and applied in tissue regeneration due to their good biocompatibility and biodegradability compared to synthetic polymeric materials. Among these natural polymeric materials, collagen and alginate have been used in the applications of bone, soft tissue, and cartilage regeneration [7,16,17,18,19]. Nevertheless, synthetic polymeric materials have been used as well due to their low cost, ease of processability, and adjustable chemical and mechanical properties. Among these different synthetic polymeric materials, polycaprolactone (PCL), poly (lactic acid/L-lactic acid) (PLA/PLLA), poly (lactic-co-glycolic) acid (PLGA), and poly (ethylene glycol) (PEG) have been widely investigated in the applications of skin, nerve, and bone regeneration [5,20,21,22]. 

Recently, natural polymeric materials have been used in combination with synthetic polymeric materials to improve bioactivity—including mechanical properties, chemical properties, and controlled chemical release for regenerative medicine [18,23,24,25,26,27]. During tissue regeneration, controllable cellular growth, connectedness, and attachment are important determining factors for the success of regeneration, which places a stronger emphasis on the need for vascularization and cellular ingress into the pores within the scaffold. A major advantage of multi-polymer-type material composition for tissue regeneration is the capability to tune mechanical strength, degradation rate, cellular adhesion, and chemical properties. For example, surface modifications have been widely explored to enhance cellular attachment and cell proliferation with different polymeric materials [28]. It has been shown in previous studies that controllable cellular growth and attachment are highly dependent on polymeric materials’ density and porosity, which depends on polymers’ mechanical strengths and physical properties [29,30]. To this end, tissue regeneration has been explored widely using a combination of natural and synthetic materials to create a porous and local bioactive environment upon implantation to regenerate damaged and injured tissues [31,32].

In the remaining sections, we summarize the different types of natural and synthetic polymeric biomaterials for the application of regenerative medicine with a focus on bioactive and biopassive polymers. Bioactivity of natural, synthetic or a combination of both biopolymers stemming from mechanical and chemical properties and controlled release of growth factors is discussed and presented in the context of their application in tissue engineering and regenerative medicine. The chapter highlights several applications including skin, bone, cartilage, and tendon regeneration.

## 2. Polymeric Materials: Naturally Derived Polymers

**Figure 1:**
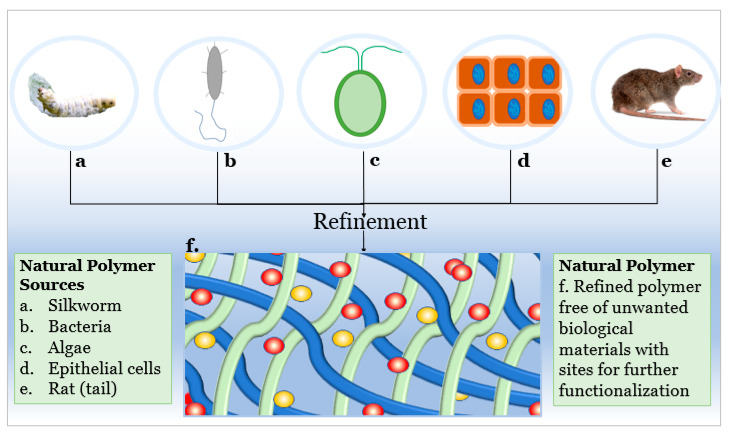
Naturally derived polymer sources: silk fibroin from silkworm (**a**), hyaluronic acid from bacteria (**b**), alginate from algae (**c**), collagen from cells and tissue (**d**), collagen from rat tail (**e**), and an illustration of a refined natural polymer showing chain organization and functionalization sites (**f**).

Naturally derived polymers, including collagen, silk, chitosan, alginate, and hyaluronic acid, are sourced from various natural systems including rat tails, epithelial cells and tissue, bacteria, algae, and silkworm (see Figure 1). The precursors to the finished polymer undergo refinement to filter out unwanted biological materials and isolate the polymer for use or so that additional modification can be made to add new functions. 

### 2.1. Collagen

Collagen is one of the most abundant proteins found in the extracellular space of musculoskeletal tissues in mammals [33]. There are more than 20 different types of collagen, which represent about 25–35% of the whole-body content. Among these different types of collagen, types I–IV are the most commonly used and have been used as scaffold materials for tissue engineering and regenerative medicine [12,33]. Recently, it has been reported that nanofibers coated with type I collagen have excellent mechanical strength and high porosity that can enhance cell attachment and proliferation, and thus can support wound healing and skin regeneration applications [34,35]. Collagen has been considered as a platform for three-dimensional (3D) culture applications instead of cells generating the substrate. For example, collagen hydrogel has been used successfully as matrices for suspending neurons and astrocytes allowing their growth and maturation and formation of neural networks in what has been alluded to as a 3D in vitro brain model [36]. Furthermore, collagen infilled 3D printed scaffolds show promise for expanding miRNA transfected progenitor cells for stimulating osteogenesis and bone repair [37]. Other collagen modification approaches can result in a mechanical property that influences the property of tissue generated. By adjusting its fibril density, with or without fibril crosslinking, its stiffness and thus the stiffness of tissue generated can be modulated as well as immune response [38,39]. 

Collagen hybridized with other extracellular matrix materials (such as alginate, fibronectin, and hyaluronic acid) also shows potential as a platform for biomechanical signal stimulation, enhanced proliferation, induction of differentiation, and the promotion of vessel endothelialization in tissue regeneration [40,41,42,43]. Moreover, the combination of collagen with other synthetic polymers has been involved in tissue regeneration applications as well. For example, Swarnalatha et al. [32] reported the application of composite PLA–collagen–chitosan enhanced cell proliferation for blood vessel outgrowth. Mechanical properties of collagen have been modulated through crosslinking to yield collagen with higher mechanical stability to benefit desired collagen hydrogel stability when implanted into skin for dermal tissue regeneration [44]. Due to its integrity and processability, fibrous collagen scaffolds have been applied in the regeneration of various tissues, including cardiovascular [15], skin [45], tendon, and ligament [46] regenerations. Despite its range of utility, host response to collagen types manifested as autoimmunity remains a concern. Type II and IV collagen are considered arthritogenic while Types I and III do not induce autoimmune reactions [47].

### 2.2. Chitosan

Chitosan, a linear polysaccharide derived from deacetylation of chitin is a biocompatible and biodegradable cationic polymer. It is the main component in the exoskeletons of crustaceans’ shells. Chitosan has a similar structure to glycosaminoglycans (GAGs), which can be degraded into different products, such as glycosylated collagen, by enzymes [48,49]. This structural similarity also enables chitosan to enhance cell adhesion compared to other synthetic biomaterials [50,51]. Chitosan is a bioactive polymeric material that has a variety of applications in tissue engineering and regenerative medicine due to its controllable mechanical properties, reactive functional groups, ease of processability and minimum foreign body rejections. Specifically, chitosan’s reactive functional groups (amino and hydroxyl moieties) provide great possibility for modifications with different entities that can result in stimuli-responsive biopolymeric materials for the applications of tissue engineering and regenerative medicine. For example, Walker et al. [52] have developed a temperature sensitive chitosan-based hydrogel to mimic the cartilage matrix for cartilage regeneration. Khan et al. [18] have demonstrated a mixed hydrogel by mixing chitosan and poly(ethylenimine) (PEI) to form pH-sensitive chitosan-PEI scaffolds for enhancing cell attachment, proliferation, and cartilage tissue engineering. Another study has revealed that the combination of chitosan and fibroblast growth factor type 2 improved wound closure and fostered the formation of granulation tissue and capillary network [53]. As an ideal natural biomaterial, chitosan itself or in combination with other hydrogels can be applied for the applications of cartilage [17,25], bone [2,23,24,54,55], nerve [56,57], and other soft tissue regenerations [16,58].

### 2.3. Alginate

Alginate is an anionic and hydrophilic polysaccharide derived primarily from marine brown seaweed or algae [59,60]. Alginate contains (1–4)-linked β-D-mannuronic acid (M) and α-L-guluronic acid (G) residues [61]. Between these two residues, G residues can form ionic crosslinks by associating with divalent cations, which can adjust the affinity of alginate. Moreover, due to its outstanding properties including ease of processability and moldability, excellent biocompatibility and biodegradability, alginate has a broad range of applications as a natural biopolymeric material, especially as three-dimensional (3D) supporting matrices for tissue restoration and regeneration. Furthermore, alginate has the ability to interact with cationic polyelectrolytes and proteoglycans, which enables alginate to form pH-dependent gels. Therefore, alginate plays an important role in the biodegradability and long-term stability as a polymeric material. For example, it has been reported by Rowley et al. [62] that the combination of alginate and other molecules could enhance cellular attachment. Furthermore, as a relatively larger polymer with a molecular weight (MW) up to 500 kDa and presence of carboxylate side groups, the degradation rate and mechanical properties of alginate-based biomaterials can be modulated. Recently, Klock et al. [63] have demonstrated an approach to form hydrogel by encapsulating alginate with cells and molecules to mimic the natural extracellular matrix (ECM) of tissues that have widespread applications in tissue regeneration. In recent studies, more and more attention has been given to the combination of alginate with other natural or synthetic polymeric materials for broader applications including bone regeneration [7], skin regeneration [64], wound healing [65], stem cell treatment [66], cartilage regeneration [19,26], and cardiac tissue regeneration [67].

### 2.4. Hyaluronic Acid

Hyaluronic acid (HA) is an anionic, nonsulfated glycosaminoglycan. It is a complex polysaccharide containing amino, hydroxyl, and carboxyl groups, allowing for its further functionalization [68]. HA is a compound already contained and utilized by the body within the connective and epithelial tissues, and drug carriers made from this material can therefore be metabolized. Partly due to this property, it has been used in drug delivery to aid wound repair, cancer treatment, inflammation control, respiratory disease treatment, and regenerative medicine [69,70,71,72]. It has also found utility in products that aid the regeneration of aged or diseased cells/tissues. An example is Hylase Wound Gel^®^ (ECR Pharmaceuticals Co. Inc., Richmond, VA, USA) which uses the salt of hyaluronic acid, sodium hyaluronate, to maintain tissue hydration and support the healing process of ulcers [68]. Several routes of hyaluronic acid administration including via ocular, nasal, pulmonary, and parenteral paths have been approved due to the sheer volume research around the HA material. Low viscosity hyaluronic acid can be used to enhance the bioavailability of ocular components due to its bioadhesive properties [68]. As such, hyaluronic acid-based eye drops for the treatment of dry eye syndrome are in preclinical evaluation. It binds to water molecules through noncovalent bonds which drive the formation of a gel-like composition. A recent study on the modification of hyaluronic acid with gelatin and Epigallocatechin gallate (EGCG), an ophthalmic pharmaceutical formulation for the treatment of dry eye syndrome, reported an enhanced retention of the medication within the eye due to hyaluronic acid’s mucoadhesive properties [68].

### 2.5. Silk Fibroin

Thousands of years ago, silkworm silk was identified as a precious material with many favorable properties. Silks are regularly defined as protein polymers produced by arthropods such as the silkworm and include others such as spiders and bees [73,74]. Silk Fibroin (SF) is the main protein derived from silkworm silk and has been introduced to the field of biomaterials for drug delivery and tissue engineering. Two SF filaments are located in the core of silk and are mostly responsible for their mechanical strength; SF has excellent tensile properties compared to other biopolymers such as collagen and higher toughness than Kevlar [73]. SF can be made into many different structures such as thin film, particulate structures, and three-dimensional structures [74]. In the biomedical field, silk film has been used as a wound dressing to treat dermatological ailments by aiding the wound healing process [74]. One exciting application of SF is its utility as bio-inks for 3D printing [72,73,74] of hydrogels to mimic the extracellular matrix to support chondrocyte and cartilage formation in vitro [74].

## 3. Polymeric Materials: Synthetically Derived Polymers

### 3.1. Polycaprolactone (PCL)

Polycaprolactone (PCL) is a semicrystalline polyester material which has been widely used for the applications of tissue engineering and regeneration due to its excellent mechanical properties [75,76]. Moreover, the thermal, mechanical, and physical properties of PCL can be modulated by mixing PCL with other natural or synthetic biomaterials, and the degradation rate depends upon molecular weight and degree of crystallinity. Compared to other biomaterials, PCL has the advantage of a high permeability, low degradation rate, excellent mechanical properties, and nontoxic byproducts, which has rendered it useful for applications of tissue regeneration in recent years. For example, Williams et al. [77] and Fujihara et al. [78] have demonstrated the applications of PCL-based scaffolds for bone regeneration; Barbarisi et al. [22] and Ranjbarvan et al. [5] have shown the applications of nerve and skin tissue regeneration by using PCL-based scaffolds. Moreover, PCL blended with other synthetic biomaterials (i.e., PLLA, PLGA) has been investigated for tissue engineering [21,79]. Due to its strong solubility and blend compatibility, PCL has been studied and used for the applications of cardiac tissue regeneration [80], vascular grafts [13], and drug delivery [20,81].

### 3.2. Poly (Lactic Acid) (PLA)

Poly (lactic acid) (PLA) is a thermoplastic and aliphatic polyester produced from nontoxic, renewable sources such as sugarcane and starch [82]. Lactide acid has two optical active isomers—L-lactide and D-lactide. Poly (L-lactic acid) (PLLA) is a semicrystalline polymer with a crystallinity of 37%. PLLA has a broader range of applications in tissue engineering and regenerative medicine due to its low degradation rate, high tensile strength, ease of processability, and nontoxic byproducts. However, due to its high crystallinity, the usage of PLLA as a scaffold material can lead to inflammation in the body. Therefore, by blending with other biomaterials or associating with other molecules, the bioactivity of PLLA-based scaffolds can be improved. For example, Cui et al. [83] have successfully demonstrated that Ginsenoside Rg3 (G-Rg3)-coated PLLA fibrous scaffolds reduced scar formation in a rabbit model. As a synthetic biopolymeric material, PLA/PLLA have been used for the applications of wound healing [84], nerve graft regeneration [85], and bone regeneration [86]. 

### 3.3. Poly (Lactide-Co-Glycolic) Acid (PLGA)

Poly (lactic-co-glycolic) acid (PLGA) is a biodegradable thermoplastic polymer synthesized by polymerization of two different monomers—L-lactic acid (LA) and glycolic acid (GA) [86]. Among a variety of different synthetic biomaterials, PLGA has been a widely used polymer for the applications of tissue engineering and regenerative medicine due to its tunable degradation rate and good mechanical properties, especially toughness, high compatibility, and excellent processability. By adjusting the ratio of LA and GA, the degradation rate can thus be modulated accordingly. The higher the ratio of GA, the faster PLGA is expected to degrade. Moreover, the mechanical properties of PLGA-based porous scaffolds have been investigated for the application of tissue engineering and regenerative medicine. It has been demonstrated that the mechanical properties of PLGA-based scaffolds can be modified by adjusting pore size, shape, porosity, and copolymer composition [31]. Due to these attractive characteristics, a variety of 3D PLGA-based scaffolds were fabricated for skin regeneration [6], cartilage regeneration [3,4], bone regeneration [9,10,87], and nerve and vascular regeneration [27,85].

### 3.4. Poly(Ethylene Glycol) (PEG)

Hydrogels are a fast developing group of polymeric materials for 3D scaffolds in the applications of tissue repair, restoration, and regeneration due to their highly swollen 3D microenvironment that mimics soft tissues and allows transportation of nutrients [88,89]. Among a variety of different types of hydrogels, PEG-based hydrogels have advantages due to their adjustable mechanical and chemical properties, capability to photopolymerize, and controllable scaffold architecture [90,91]. PEG is a hydrophilic polymer that can have a high water content when crosslinked into networks. In addition, due to the flexible and neutrally charged surface, PEG has the properties of low toxicity, low protein adsorption, and nonimmunogenicity. The chemical and mechanical properties can thus be modified by adjusting the molecular weight, initiator, and geometries to have a varied range of applications [92,93]. In recent years, PEG-based hydrogels have been used for a variety of tissue regenerations, including cartilage tissue [94], bone tissue [11], neural tissue [95], microvasculature formation [14], and cornea tissue [96].

## 4. Advantages and Disadvantages of Naturally Derived Polymers

Naturally derived polymers have excellent properties in the areas of immunogenic response and biocompatibility. One of the reasons for naturally derived polymers’ excellent biocompatibility properties is the possession of known cell-binding sites [97,98]. These sites allow for the present growth factors to help the regeneration of the desired tissue [98]. One example of this is the compatibility of fibroblast attachment to collagen composite scaffolds for over 14 days postcellular seeding [98]. Since these polymers are naturally derived and contain many natural extracellular matrix components, the bodily immune response is minimal compared to synthetic polymers [98].

Although naturally derived polymers have advantages of low immunogenic response and biocompatibility, they lack other crucial properties. One drawback of naturally derived polymers is the limited ability to tailor the polymers for specific properties such as mechanical properties and porosity [98]. When making scaffolds for tissue engineering purposes, tailoring pore size and mechanical properties for different cell types are necessary to improve the viability and proliferation of the desired tissue. Naturally derived polymers are subject to batch-to-batch variations, including an inaccurate mixture of biological factors, leading to difficulty in repeatability of experiments and studies, which is not desirable [99]. Since natural polymers are derived from living tissue or from excretions of specific animals, the supply is limited, which increases the price of naturally derived polymers, being significantly more expensive than synthetically derived polymers [97].

## 5. Advantages and Disadvantages of Synthetically Derived Polymers

Synthetic polymers derived from chemical methods or processes allow for different structures and molecular weights to be synthesized from the polymerization of different monomers. With versatility in functional groups that can be added onto polymer backbones and conjugation of different natural macromolecules and proteins onto the synthetic polymer, multifunctional hybrid materials that exhibit desirable properties such as biocompatibility, biodegradability, and triggering of intracellular signal transduction can be engineered into scaffolds [100]. Synthetic polymers are cheaper and can be designed to cover a range of properties enabling their use as appropriate substitutes for naturally derived polymers. PCL, for example, has a wide range of synthesis conditions which allow for different mechanical properties to be designed into scaffolds, as well as functional activities such as the promotion of cell adhesion, growth, and differentiation, and even as a transport vector for bioactive molecules [101].

On the other hand, synthetic polymers lack the biological cues found in the ECMs of naturally derived polymers and can induce immune responses without appropriate modifications [102]. The byproducts from the degradation of synthetic polymers within the host can also cause several problems, including acid accumulation and inflammation [100,103]. For example, a gradual accumulation of acidic byproducts from PLGA substrates in bone-derived mesenchymal stem cells culture led to acidification and demineralization of the culture medium [104].

## 6. Applications of Polymeric Materials in Regenerative Medicine

### 6.1. Mechanical and Porosity Effects on Tissue Engineering Scaffolding Functions

Tissue engineering scaffolds’ primary function is to mimic the desired tissue’s extracellular matrix and additionally to increase cell proliferation and viability [105,106]. The correct materials need to be applied to best match the tissue’s extracellular matrix. Mechanical properties of the scaffolding are essential to provide the right environment for the tissue. For example, Griffith et al. developed a collagen sponge used to fabricate a scaffold for a tissue engineered cornea. The collagen-chondroitin sulfate-based hydrogel was modeled to match the tissue’s extracellular matrix in the cornea [107]. Layers of human corneal epithelial and endothelial cells were used to mimic the exact tissue in the cornea. Griffith et al. were successful in representing a typical human cornea due to modification of the scaffolding to increase tensile strength from 800 to 1900 kPa [107].

Pores can supply valid space for cell attachment or restrict cytokines and chemokines. Porosity, the ratio of the void space to that of the solid, and pore connectedness can influence cell kinetics and tissue ingrowth as well as the mechanical property of the polymer scaffold [108]. For example, primary rat osteoblast migrated faster in PolyHIPE polymer foam with larger pores (100 nm) compared to smaller pores [109].

The ideal scaffold should have consistent mechanical properties and porosity that match the mechanical property of the tissue to be engineered and support biological kinetics [110]. The elastic modulus, tensile strength, and fatigue should match the corresponding tissue to enhance tissue compatibility. For example, in a study to match polymer scaffold’s mechanical property to cancellous spongy bone compressive strength (1.9 to 7.0 MPa); chitosan and hydroxyapatite nanoparticles were used to form a honeycomb-structure scaffold [111,112] which yielded a high porosity and similar compressive strength (1.62 ± 0.22 MPa). This supported the proliferation of MC3T3-E1 cells on the scaffold and that parameters that drive the attainment of mechanical integrity of the new tissue and angiogenesis through porous structures are important elements of scaffold design.

### 6.2. Regenerative Medicine

The question now is how far have tissue regeneration technologies have advanced and what roles do the above polymers play in those technologies? Key opinion leaders in biomaterials research for various medical applications, including long-term drug release from polymers and tissue engineering or regenerative medicine, claim that it is a matter of when and not if polymers will be used in the generation of new tissues and organs. Today, we can already create new skin for burn victims, a technology that is being used in the clinics. There are other organ and tissue engineering technologies in clinical trials that rely on knowledge accumulated over the 30 years since tissue engineering work began. Biomaterials of all types are being used across several tissue and organ regeneration methods and to develop tissue engineering to support people with debilitating medical conditions such as liver dysfunction, damaged tissue from traumatic injuries, or seriously damaged organs. Polymers make it possible for the creation of multidimensional platforms or scaffolds that support the early stages of the development of cells into tissue by serving either as permanent or pro tem artificial extracellular matrices. Pro tem artificial ECMs may be nonbiodegradable, especially when used as supports in vitro while biodegradable versions are desirable for in vivo applications [113,114]. Investigations of polymeric materials in regenerative medicine have increased over the decades, with their application in bone tissue being the most researched area [115]. On the other hand, the use of biomaterials in tissue regeneration investigations for skin, cartilage, and tendon generation have either been sustained or increased over the years.

Examples of regenerative applications using biomaterials include regeneration of cartilage, skin, heart, lung, valves, vascular grafts, kidney, liver, pancreas, bladder, bone cement, and artificial blood vessels [116,117,118,119,120,121,122,123,124,125,126,127,128,129,130,131,132,133]. A selection of these applications will be discussed below focusing on the biomaterial(s) typically used for each technology, their approval by regulatory bodies, stage of development of the technology, and current challenges.

### 6.3. Skin Regeneration

Skin regeneration via tissue engineering has achieved success in the clinical setting in recent years through the use of engineered polymers that promote wound healing. Several polymers including fibrin and polyesters (e.g., poly 3-hydroxybutyrate) used as support materials towards this goal are currently under investigation, although collagen-based polymer scaffolds have increasingly been shown to be comparably effective. For example, a collagen-based scaffold developed in the form of wound grafts, has received approval from the U.S. Food and Drug Administration and is now marketed as Integra. Other approved collagen-based materials include Collaplug^®^ (Zimmer Biomet, Warsaw, IN, USA) and Ultrafoam^®^ (Becton Dickinson, Franklin Lakes, NJ, USA). Type I collagen is the most abundant collagen and it is a natural polymer that is part of the extracellular matrix, a structural material which provides shape and form to tissue. They form extensive interconnecting fiber networks which provide physical support as well as supply mechanical and chemical cues to adherent cells.

**Figure 2:**
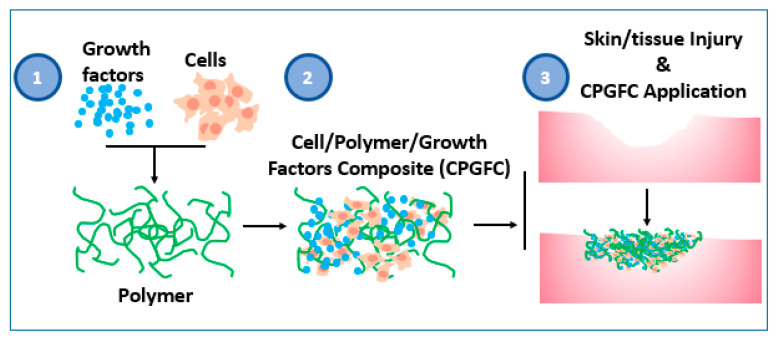
General approach of polymer-based methods for tissue regeneration typically involving application of a composite of biodegradable natural or synthetic polymeric matrices, growth factors, and seeding cells to the damaged tissue site. In step (**1**) growth factors and cells are seeded in polymer or cells are seeded on growth factor conjugated polymer; in (**2**) nutrients, oxygen, and appropriate physiological support are supplied to CPGFC in vitro for conditioning before application in vivo or may be applied directly without conditioning; and in (**3**) site of the tissue injury is dressed with bio-functional CPGFC composite materials.

As shown in Figure 2, the typical composition of these tissue regeneration templates will comprise the polymeric material or substrate that supports seeding cells and growth factor immobilization. The substrates may or may not be degradable and are designed to be nontoxic to cells, promote integration of surrounding cells, and for controlled release of their growth factors.

The application of Integra in severely injured burned adult patients has been associated with decreased length of hospital stay. In a survival and length of hospital stay retrospective study conducted by Ryan CM et al. [134] using Integra dermal regeneration material in over 270 adults with burn sizes not less than 20% of body surface area, no difference in mortality was observed between groups who received Integra dermal regeneration treatment and those who did not. It was found that the length of hospital stay in older (>60) patients with two or more mortality risk factors and with burn sizes greater than 40% of body surface area was shorter (63 days) compared to 107 days in patients with two or more risk factors who did not receive Integra.

### 6.4. Cartilage Regeneration

Articular cartilage material is composed of water, electrolytes, collagen, proteoglycans, glycoproteins, other proteins, and chondrocytes. Defects in articular cartilage are common in orthopedic practice, but current treatment outcomes are only generally positive in the short-term while long-term results vary. Repopulation of cartilage defects are being carried out using hyaline cartilage polymers which contain living chondrocytes for injection at the defect site through a minimally invasive surgical procedure to improve clinical outcomes of conventional procedures including microfracture, physiotherapy, arthroscopic chondroplasty, or autologous chondrocyte implantation. Hyaline cartilage is the most abundant type of cartilage and is found inside bones and in the lining between bone joints. Bone growth or ossification originates from these materials. On the other hand, collagen, agarose, starch, poly(lactic acid), hyaluronic acid, poly(glycolic acid)/poly(lactic-co-glycolic acid) (PGA/PLGA), poly(N-isopropylacrylamide) (poly(NIPAAm)), and poly(propylene fumarate) (PPF) are among other natural and synthetic polymers that have been applied to cartilage tissue regeneration.

Desirable properties of these polymers are their formation of hydrogels in situ and hydrophilicity. They can therefore be injected to form scaffolds in situ so complex surgical procedures may be avoided. An example of an injectable hydrogel is chitosan and hyaluronic acid blend with encapsulated chondrocytes; this composite has shown promise in cartilage repair [135]. Chemically crosslinked chitosan particles have also been proposed as injectable microparticles [136]. Degradation of the hydrogels is an important fabrication parameter and can influence outcomes of tissue regeneration. Ideally, they must dissolve away as cartilage tissue is being formed and to allow total ingrowth of the new tissue. Too slow a degradation rate may unnecessarily delay healing and too fast a degradation rate may lead to incomplete or no healing. Alginate, for example, has desirable biocompatibility properties and can form hydrogels, but its slow and difficult to control degradation can lead to undesirable tissue regeneration outcomes. Alginate has been reported to remain almost fully present after 3 months of implantation for cartilage regeneration although newly formed tissue was histologically normal [137].

### 6.5. Bone Regeneration

Bone regeneration is a physiological process of bone formation that is initiated to heal fractures and continuously remodel skeletal tissue with age. In conditions such as large bone fracture, severe and widespread infection of the bone, osteoporosis, and nonunions, the normal bone formation process becomes inadequate. In such cases, technologies including autologous bone graft, allograft implantation, osteoconductive scaffolds, bone growth factors, and osteogenitor cell implantation are being used to address these defects. 

However, more localized approaches have recently been pursued to address the shortcomings of the aforementioned remediation options to accelerate the healing process and to produce graft substitutes with appropriate mechanical properties. These approaches are supported by a large number of synthetic bone substitutes including hydroxyapatite, β-tricalcium phosphate, and calcium-phosphate cements, and glass ceramics [138,139] which are used to promote bone cell kinesis, proliferation, and differentiation for bone regeneration. These substitutes circumvent immunogenicity and rejection reactions, the possibility of infection transmission, and cost issues associated with allogenic bone grafts [140,141] as well as the surgical harvesting procedure and its complications, quantity restriction, and surgical cost associated with autograph grafts [142,143].

Commercially available collagen-based polymers for bone regeneration include collagen bone healing protective sheets derived from type I collagen, Collaplug^®^ (Zimmer Biomet, Warsaw, IN, USA), and Ultrafoam^®^ (Becton Dickinson, Franklin Lakes, NJ, USA). 

Currently, no heterologus or artificial bone substitutes have the same mechanical and biological properties as bone; hence, alternative approaches whether used adjunctively or as alternatives remain the goal in bone regeneration. These alternatives must overcome current challenges and adequately capture the temporal and spatial properties of the bone formation process. Recent advances in cellular and molecular and cellular biology keep elucidating genes which encode for proteins that play key roles in bone repair [143] and such discoveries may help advance the field of regenerative medicine.

### 6.6. Tendon Regeneration

Tendons connect skeletal muscles to bones and their complex structures and unique mechanical properties present difficult challenges for developing scaffolds that adequately mimic these physical attributes. They are made up of multiple layers of connective tissue consisting mainly of collagen fibers and the tendon tissue transitions, on one end, to connect with muscles at the myotendinous junction and on the other end to connect to compact bone at the osteotendinous junction. Due to this complexity, only few natural and synthetic polymer scaffolds have been proposed for tendon regeneration. 

As collagen and polysaccharides are among the major components of the natural tendon extracellular matrix which support cell adhesion and proliferation, they are among the natural polymers being used for tendon tissue regeneration. Concerns with these materials are their different mechanical properties to tendons that they present and the difficulty in their processing. Other natural polymers, including chitosan and chitin, are being investigated for tendon tissue regeneration as well. 

For synthetic polymers, PGA, PLA, and polyesters are being studied for tendon tissue regeneration. Because they are synthetic materials, they present the ability to engineer them for desired mechanical properties. For those that are biodegradable, their byproducts may interact with biology either positively or can lead to undesirable effects. For example, PGA and PLA will biodegrade into glycolic and lactic acids, which are natural metabolites, and thus are biocompatible in moderate quantities [144,145,146].

Tendon tissue regeneration products in clinical use include the commercial scaffolds: GraftJacket© (Wright Medical, Memphis, TN, USA), Restore™ (DePuy Orthopedics, Warsaw, IN, USA), TissueMend© (Stryker Orthopedics, Mahwah, NJ, USA), CuffPatch© (Arthrotek, Warsaw, IN, USA), Zimmer patch, formerly known as Permacol™ (Zimmer, Warsaw, IN, USA), Shelhigh No-React© Encuff Patch (Shelhigh Inc., Union, NJ, USA), OrthADAPT© (Pegasus Biologic Inc., Irvine, CA, USA), Bio-Blanket© (Kensey Nash Corp., Exton, PA, USA), Gore-Tex© patch WL (Gore and Associates, Flagstaff, AZ, USA), Lars© ligament (Dijon, France), Leeds–Keio© or Poly-tape© (Xiros plc, Neoligaments, Leeds, UK; Yufu Itonaga Co., Ltd., Tokyo, Japan) and Artelon© & Sportmesh™ (Artimplant AB, Västra Frölunda Sweden & Biomet Sports Medicine, Warsaw, IN, USA) [144]. The first eight are sourced from animal and human cadavers while the remainder are made of synthetic materials. Their clinical outcomes so far have been generally inconsistent and seem to depend on the tendon involved—be it the rotator cuff, Achilles, or trapeziometacarpal. Current data seem to suggest rotator cuff tendon regeneration applications have been less successful than Achilles tendon procedures, although this may be because more rotator cuff procedures are performed and hence the number of reported failed procedures is higher [147].

## 7. Outlook

Polymers used to support tissue regeneration, whether in the form of a scaffold or not, are designed to assist the restoration of tissues or organs to normal function and may either serve as a pro tem material that degrades away after a period of time or permanently remains at the implant site. These materials must therefore have desirable physical and chemical properties that effectively aid the regeneration of tissue involved. It must present the desired mechanical strength to sustain load bearing needs so neighboring healthy tissues are protected. It must aid tissue ingrowth and accelerate regeneration and also be biocompatible. 

A deeper understanding of the roles of growth factors, small molecules, peptides, and oligonucleotides and their stable incorporation into these materials as well as their controlled release are important for advancing the field. Thus, guiding of development of next generation scaffold materials with design principles and modification processes (see Table 1) that allow for, among other things, the desired outcomes, including controlled drug delivery, greater ingrowth of stem cell-derived cells or of genetically modified cells which amplify key proteins for tissue regeneration may translate our current technologies into ones with polymeric materials/scaffolds with improved safety and efficacy of bioactivities to accelerate tissue regeneration for better patient outcomes. 

## Figures and Tables

**Table 1:** Widely accepted and emerging biopolymer structure–property relationships.

Biopolymer (Highlighted Groups Are Modification Sites)	Material Bioactivity	Experimental Stage Modifications for Additional Bioactivity
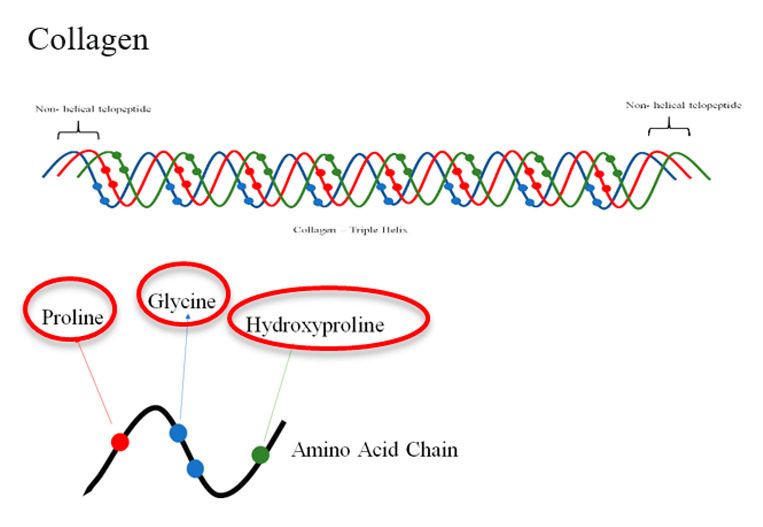	Integrin-binding motif on collagen allows the anchorage of cells through transmembrane integrins Carbonyl functional groups susceptible to hydrolysis [148]	Bifunctional polyacrylic acid/N-hydroxysuccinimide (PAA-NHS) crosslinker for conjugating collagen’s -COOH and –NH_2_ groups to align with –COOH and –NH_2_ groups on PAA-NHS to increase solubility and thermal stability of collagen [149]
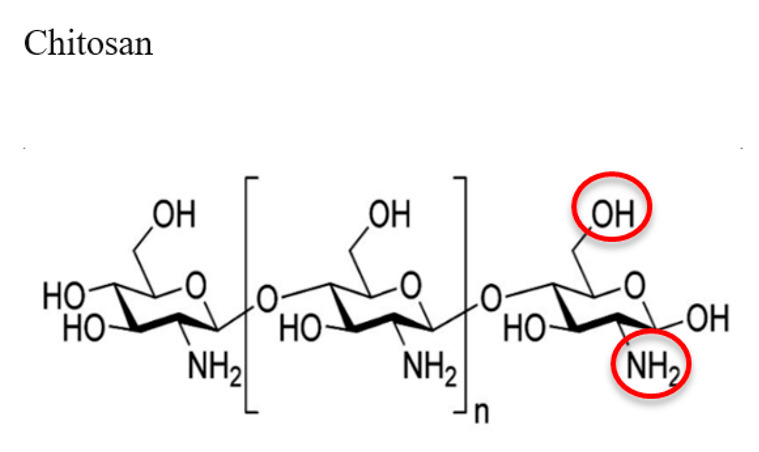	Soluble in water (also blood plasma) Biodegradable Binds to negatively charged surfaces (cell membranes) Has several hydrogen bonding sites (hydroxyl groups and carbonyl oxygens) for protein binding [150,151]	Direct reaction of NO biomolecule with amides on chitosan to form diazeniumdiolates (in this case, a chitosan NO donor for cell signaling, proliferation, and protective outcomes) Hydroxyl and amine groups are also functionalizable via alkylation (addition of hydrocarbons to increase hydrophobicity) Hydroxyl groups can be thiolated (addition of –SH group) followed by bioconjugation EDC can be reacted to –COOH functional groups on proteins and polymers to form complexes for conjugation to primary amines on chitosan
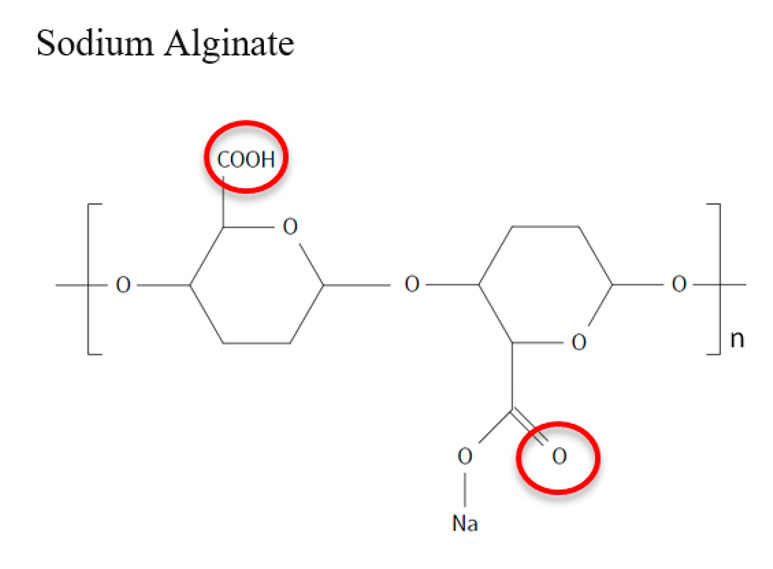	Carbonyl functional group susceptible to hydrolysis Gelation in the presence of cations	Hydroxyl and carboxyl groups are sites for hydrogen bonding and chemical modifications Hydroxyls have been modified by oxidation, leading to increased degradation rate of alginate (drug delivery implications), Oxidized alginate can further be modified with amines by reductive-amination to exhibit amphiphilic properties Modification by sulfation to form alginate sulfates with structural likeness to heparin and with an anticoagulation property Carboxyl groups have been modified by esterification (attaching alkyl groups) to increase the hydrophobicity of alginate. See Ref. 152 for other modifications [152]
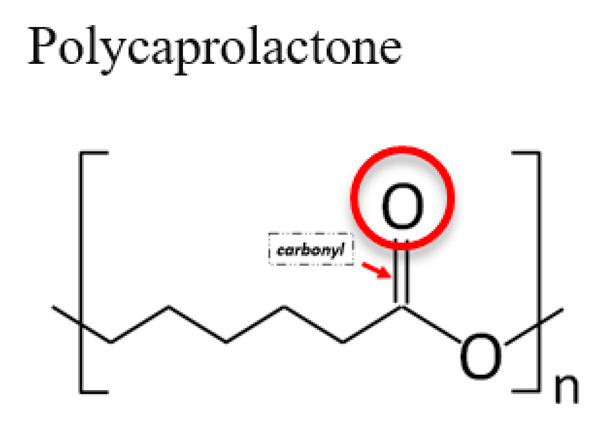	Carbonyl functional group susceptible to hydrolysis Hydrogen bonding	Covalent attachment of Amino (–NH_2_) group on PC by reacting 1,6-hexanediamine with ester groups in PC and proteins can be attached to their free amines Nanotopography formation on PC from solvent treatment to influence cell adhesion [153,154]
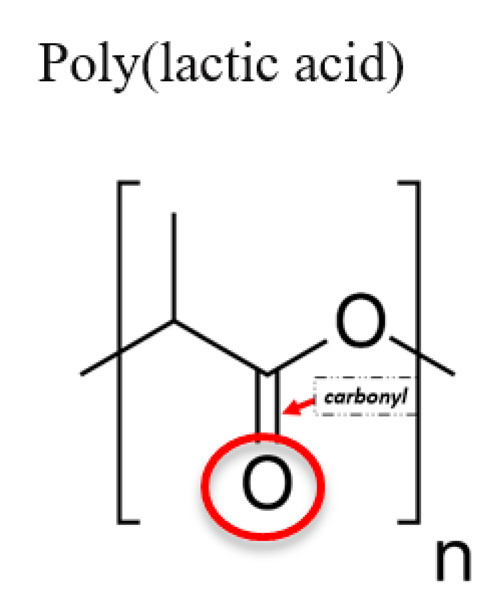	Biodegradable (carbonyl group susceptible to hydrolysis) Hydrogen bonding	Co-polymerization with polyesters (poly(glycolide), poly(epsilon-caprolactone), poly(beta-hydroxybutyrate), etc.) for new material properties and with polysaccharides for faster hydrolytic degradation Alkaline surface hydrolysis for creating carboxylic acids (–COOH) and hydroxyl (–OH) groups on PLA, which can then be conjugated with surface modifying species containing amine (–NH_2_) of hydroxyl (–OH) groups [155,156]
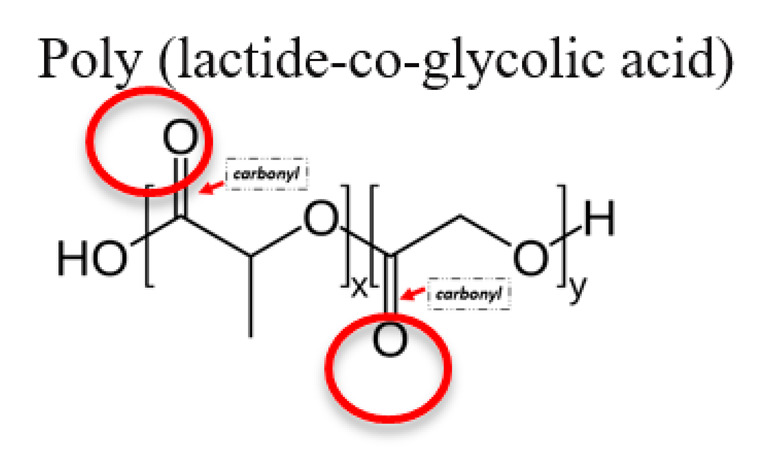	PLGA is a biodegradable polymer, and its degradation rate can be modulated with glycolic amounts relative to lactide. Carbonyl functional groups susceptible to hydrolysis	Carbodiimide conjugation chemistry can be used to attach different moieties like alendronate in order to target specific sites. Alendronate is a molecule with a higher affinity to bone tissue so, with this material, it could be possible to target an active compound such as N-acetylcysteine to bone tissue [157,158]
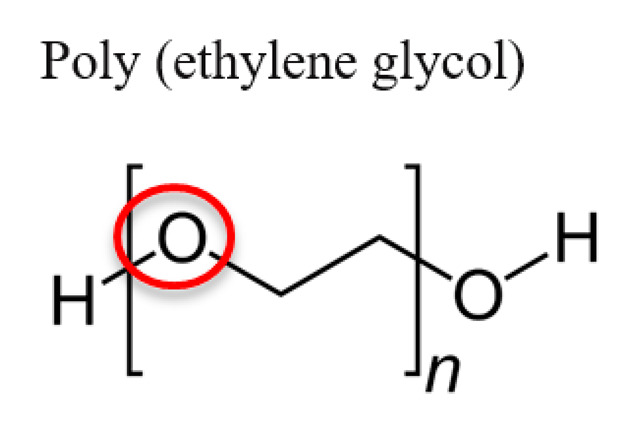	PEG is hydrophilic and amphiphilic (hydrophilic and lipophilic) [159]	Covalent conjugation of N-hydroxysuccinimide (NHS)-terminated PEG to amines on protein for improved protein pharmacokinetics (in clinical use) or to peptides, small molecules, and oligonucleotides [159,160]

## Data Availability

Data sharing is not applicable to this article.

## References

[1] Mao A.S., Mooney D.J. (2015). Regenerative medicine: Current therapies and future directions. Proc. Natl. Acad. Sci. USA.

[2] Beck S., Jiang T., Nair L., Laurencin C. (2017). Chitosan bone and cartilage for regenerative engineering. Chitosan Based Biomaterials.

[3] Lin W., Liu Z., Kampf N., Klein J. (2020). The Role of Hyaluronic Acid in Cartilage Boundary Lubrication. Cells.

[4] Mouthuy P.A., El-Sherbini Y., Cui Z., Ye H. (2016). Layering PLGA-based electrospun membranes and cell sheets for engineering cartilage–bone transition. J. Tissue Eng. Regen. Med..

[5] Ranjbarvan P., Soleimani M., Samadi Kuchaksaraei A., Ai J., Faridi Majidi R., Verdi J. (2017). Skin regeneration stimulation: The role of PCL-platelet gel nanofibrous scaffold. Microsc. Res. Tech..

[6] Kumbar S.G., Nukavarapu S.P., James R., Nair L.S., Laurencin C.T. (2008). Electrospun poly (lactic acid-co-glycolic acid) scaffolds for skin tissue engineering. Biomaterials.

[7] Venkatesan J., Bhatnagar I., Manivasagan P., Kang K.-H., Kim S.-K. (2015). Alginate composites for bone tissue engineering: A review. Int. J. Biol. Macromol..

[8] Gregor A., Filová E., Novák M., Kronek J., Chlup H., Buzgo M., Blahnová V., Lukášová V., Bartoš M., Nečas A. (2017). Designing of PLA scaffolds for bone tissue replacement fabricated by ordinary commercial 3D printer. J. Biol. Eng..

[9] Geuze R.E., Theyse L.F., Kempen D.H., Hazewinkel H.A., Kraak H.Y., Öner F.C., Dhert W.J., Alblas J. (2012). A differential effect of bone morphogenetic protein-2 and vascular endothelial growth factor release timing on osteogenesis at ectopic and orthotopic sites in a large-animal model. Tissue Eng. Part A.

[10] Sicchieri L.G., Crippa G.E., de Oliveira P.T., Beloti M.M., Rosa A.L. (2012). Pore size regulates cell and tissue interactions with PLGA–CaP scaffolds used for bone engineering. J. Tissue Eng. Regen. Med..

[11] Burdick J.A., Anseth K.S. (2002). Photoencapsulation of osteoblasts in injectable RGD-modified PEG hydrogels for bone tissue engineering. Biomaterials.

[12] Aravamudhan A., Ramos D.M., Nip J., Harmon M.D., James R., Deng M., Laurencin C.T., Yu X., Kumbar S.G. (2013). Cellulose and collagen derived micro-nano structured scaffolds for bone tissue engineering. J. Biomed. Nanotechnol..

[13] Wang Z., Cui Y., Wang J., Yang X., Wu Y., Wang K., Gao X., Li D., Li Y., Zheng X.L. (2014). The effect of thick fibers and large pores of electrospun poly (ε-caprolactone) vascular grafts on macrophage polarization and arterial regeneration. Biomaterials.

[14] Leslie-Barbick J.E., Saik J.E., Gould D.J., Dickinson M.E., West J.L. (2011). The promotion of microvasculature formation in poly (ethylene glycol) diacrylate hydrogels by an immobilized VEGF-mimetic peptide. Biomaterials.

[15] Sell S.A., McClure M.J., Garg K., Wolfe P.S., Bowlin G.L. (2009). Electrospinning of collagen/biopolymers for regenerative medicine and cardiovascular tissue engineering. Adv. Drug Deliv. Rev..

[16] Cheung H.K., Han T.T.Y., Marecak D.M., Watkins J.F., Amsden B.G., Flynn L.E. (2014). Composite hydrogel scaffolds incorporating decellularized adipose tissue for soft tissue engineering with adipose-derived stem cells. Biomaterials.

[17] Naderi-Meshkin H., Andreas K., Matin M.M., Sittinger M., Bidkhori H.R., Ahmadiankia N., Bahrami A.R., Ringe J. (2014). Chitosan-based injectable hydrogel as a promising in situ forming scaffold for cartilage tissue engineering. Cell Biol. Int..

[18] Khan F., Tare R.S., Oreffo R.O., Bradley M. (2009). Versatile biocompatible polymer hydrogels: Scaffolds for cell growth. Angew. Chem..

[19] Ghidoni I., Chlapanidas T., Bucco M., Crovato F., Marazzi M., Vigo D., Torre M.L., Faustini M. (2008). Alginate cell encapsulation: New advances in reproduction and cartilage regenerative medicine. Cytotechnology.

[20] Yin H., Gong C., Shi S., Liu X., Wei Y., Qian Z. (2010). Toxicity evaluation of biodegradable and thermosensitive PEG-PCL-PEG hydrogel as a potential in situ sustained ophthalmic drug delivery system. J. Biomed. Mater. Res. Part B Appl. Biomater..

[21] Hiep N.T., Lee B.-T. (2010). Electro-spinning of PLGA/PCL blends for tissue engineering and their biocompatibility. J. Mater. Sci. Mater. Med..

[22] Barbarisi M., Marino G., Armenia E., Vincenzo Q., Rosso F., Porcelli M., Barbarisi A. (2015). Use of polycaprolactone (PCL) as scaffolds for the regeneration of nerve tissue. J. Biomed. Mater. Res. Part A.

[23] Dessi M., Borzacchiello A., Mohamed T.H., Abdel-Fattah W.I., Ambrosio L. (2013). Novel biomimetic thermosensitive β-tricalcium phosphate/chitosan-based hydrogels for bone tissue engineering. J. Biomed. Mater. Res. Part A.

[24] Niranjan R., Koushik C., Saravanan S., Moorthi A., Vairamani M., Selvamurugan N. (2013). A novel injectable temperature-sensitive zinc doped chitosan/β-glycerophosphate hydrogel for bone tissue engineering. Int. J. Biol. Macromol..

[25] Sá-Lima H., Caridade S.G., Mano J.F., Reis R.L. (2010). Stimuli-responsive chitosan-starch injectable hydrogels combined with encapsulated adipose-derived stromal cells for articular cartilage regeneration. Soft Matter.

[26] Marsich E., Borgogna M., Donati I., Mozetic P., Strand B.L., Salvador S.G., Vittur F., Paoletti S. (2008). Alginate/lactose-modified chitosan hydrogels: A bioactive biomaterial for chondrocyte encapsulation. J. Biomed. Mater. Res. Part A.

[27] Han J., Lazarovici P., Pomerantz C., Chen X., Wei Y., Lelkes P.I. (2010). Co-electrospun blends of PLGA, gelatin, and elastin as potential nonthrombogenic scaffolds for vascular tissue engineering. Biomacromolecules.

[28] Liu X., Holzwarth J.M., Ma P.X. (2012). Functionalized synthetic biodegradable polymer scaffolds for tissue engineering. Macromol. Biosci..

[29] O’Brien F.J., Harley B., Yannas I.V., Gibson L.J. (2005). The effect of pore size on cell adhesion in collagen-GAG scaffolds. Biomaterials.

[30] Zeltinger J., Sherwood J.K., Graham D.A., Müeller R., Griffith L.G. (2001). Effect of pore size and void fraction on cellular adhesion, proliferation, and matrix deposition. Tissue Eng..

[31] Pan Z., Ding J. (2012). Poly (lactide-co-glycolide) porous scaffolds for tissue engineering and regenerative medicine. Interface Focus.

[32] Swarnalatha B., Nair S.L., Shalumon K.T., Milbauer L.C., Jayakumar R., Paul-Prasanth B., Menon K.K., Hebbel R.P., Somani A., Nair S.V. (2013). Poly (lactic acid)–chitosan–collagen composite nanofibers as substrates for blood outgrowth endothelial cells. Int. J. Biol. Macromol..

[33] Lee C.H., Singla A., Lee Y. (2001). Biomedical applications of collagen. Int. J. Pharm..

[34] Rho K.S., Jeong L., Lee G., Seo B.M., Park Y.J., Hong S.D., Roh S., Cho J.J., Park W.H., Min B.M. (2006). Electrospinning of collagen nanofibers: Effects on the behavior of normal human keratinocytes and early-stage wound healing. Biomaterials.

[35] Willerth S.M., Sakiyama-Elbert S.E. (2007). Approaches to neural tissue engineering using scaffolds for drug delivery. Adv. Drug Deliv. Rev..

[36] Soscia D.A., Lam D., Tooker A.C., Enright H.A., Triplett M., Karande P., Peters S.K., Sales A.P., Wheeler E.K., Fischer N.O. (2020). A flexible 3-dimensional microelectrode array for in vitro brain models. Lab A Chip.

[37] Moncal K.K., Aydin R.S., Abu-Laban M., Heo D.N., Rizk E., Tucker S.M., Lewis G.S., Hayes D., Ozbolat I.T. (2019). Collagen-infilled 3D printed scaffolds loaded with miR-148b-transfected bone marrow stem cells improve calvarial bone regeneration in rats. Mater. Sci. Eng. C.

[38] Franke K., Sapudom J., Kalbitzer L., Anderegg U., Pompe T. (2014). Topologically defined composites of collagen types I and V as in vitro cell culture scaffolds. Acta Biomater..

[39] Sapudom J., Mohamed W.K.E., Garcia-Sabaté A., Alatoom A., Karaman S., Mahtani N., Teo J.C.M. (2020). Collagen Fibril Density Modulates Macrophage Activation and Cellular Functions during Tissue Repair. Bioengineering.

[40] Liu C., Lewin Mejia D., Chiang B., Luker K.E., Luker G.D. (2018). Hybrid collagen alginate hydrogel as a platform for 3D tumor spheroid invasion. Acta Biomater..

[41] Xiao L., Ding M., Saadoon O., Vess E., Fernandez A., Zhao P., Jin L., Li X. (2016). A novel culture platform for fast proliferation of human annulus fibrosus cells. Cell Tissue Res..

[42] Yang L., Ge L., Zhou Q., Mokabber T., Pei Y., Bron R., Rijn P. (2020). Biomimetic Multiscale Hierarchical Topography Enhances Osteogenic Differentiation of Human Mesenchymal Stem Cells. Adv. Mater. Interfaces.

[43] Kang L., Jia W., Li M., Wang Q., Wang C., Liu Y., Wang X., Jin L., Jiang J., Gu G. (2019). Hyaluronic acid oligosaccharide-modified collagen nanofibers as vascular tissue-engineered scaffold for promoting endothelial cell proliferation. Carbohydr. Polym..

[44] Lotz C., Schmid F.F., Oechsle E., Monaghan M.G., Walles H., Groeber-Becker F. (2017). Cross-linked Collagen Hydrogel Matrix Resisting Contraction To Facilitate Full-Thickness Skin Equivalents. ACS Appl. Mater. Interfaces.

[45] Liu Y., Ma L., Gao C. (2012). Facile fabrication of the glutaraldehyde cross-linked collagen/chitosan porous scaffold for skin tissue engineering. Mater. Sci. Eng. C.

[46] Kew S.J., Gwynne J.H., Enea D., Abu-Rub M., Pandit A., Zeugolis D., Brooks R.A., Rushton N., Best S.M., Cameron R.E. (2011). Regeneration and repair of tendon and ligament tissue using collagen fibre biomaterials. Acta Biomater..

[47] Lynn A.K., Yannas I.V., Bonfield W. (2004). Antigenicity and immunogenicity of collagen. J. Biomed. Mater. Res..

[48] Lim S.M., Song D.K., Oh S.H., Lee-Yoon D.S., Bae E.H., Lee J.H. (2008). In vitro and in vivo degradation behavior of acetylated chitosan porous beads. J. Biomater. Sci. Polym. Ed..

[49] Heinemann C., Heinemann S., Lode A., Bernhardt A., Worch H., Hanke T. (2009). In vitro evaluation of textile chitosan scaffolds for tissue engineering using human bone marrow stromal cells. Biomacromolecules.

[50] Custódio C.A., Alves C., Reis R., Mano J. (2010). Immobilization of fibronectin in chitosan substrates improves cell adhesion and proliferation. J. Tissue Eng. Regen. Med..

[51] Walker K.J., Madihally S.V. (2015). Anisotropic temperature sensitive chitosan-based injectable hydrogels mimicking cartilage matrix. J. Biomed. Mater. Res. Part B Appl. Biomater..

[52] Obara K., Ishihara M., Ishizuka T., Fujita M., Ozeki Y., Maehara T., Saito Y., Yura H., Matsui T., Hattori H. (2003). Photocrosslinkable chitosan hydrogel containing fibroblast growth factor-2 stimulates wound healing in healing-impaired db/db mice. Biomaterials.

[53] Wang L., Stegemann J.P. (2010). Thermogelling chitosan and collagen composite hydrogels initiated with β-glycerophosphate for bone tissue engineering. Biomaterials.

[54] Tian M., Yang Z., Kuwahara K., Nimni M.E., Wan C., Han B. (2012). Delivery of demineralized bone matrix powder using a thermogelling chitosan carrier. Acta Biomater..

[55] Valmikinathan C.M., Mukhatyar V.J., Jain A., Karumbaiah L., Dasari M., Bellamkonda R.V. (2012). Photocrosslinkable chitosan based hydrogels for neural tissue engineering. Soft Matter.

[56] Mekhail M., Almazan G., Tabrizian M. (2015). Purine-crosslinked injectable chitosan sponges promote oligodendrocyte progenitor cells’ attachment and differentiation. Biomater. Sci..

[57] Li X., Ma X., Fan D., Zhu C. (2012). New suitable for tissue reconstruction injectable chitosan/collagen-based hydrogels. Soft Matter.

[58] Skjåk-Bræk G., Grasdalen H., Smidsrød O. (1989). Inhomogeneous polysaccharide ionic gels. Carbohydr. Polym..

[59] Narayanan R.P., Melman G., Letourneau N.J., Mendelson N.L., Melman A. (2012). Photodegradable iron (III) cross-linked alginate gels. Biomacromolecules.

[60] Lee K.Y., Mooney D.J. (2012). Alginate: Properties and biomedical applications. Prog. Polym. Sci..

[61] Rowley J.A., Madlambayan G., Mooney D.J. (1999). Alginate hydrogels as synthetic extracellular matrix materials. Biomaterials.

[62] Klöck G., Pfeffermann A., Ryser C., Gröhn P., Kuttler B., Hahn H.J., Zimmermann U. (1997). Biocompatibility of mannuronic acid-rich alginates. Biomaterials.

[63] Hashimoto T., Suzuki Y., Tanihara M., Kakimaru Y., Suzuki K. (2004). Development of alginate wound dressings linked with hybrid peptides derived from laminin and elastin. Biomaterials.

[64] Vowden P., Romanelli M., Peter R., Boström Å., Josefsson A., Stege H. (2006). The effect of amelogenins (Xelma™) on hard-to-heal venous leg ulcers. Wound Repair Regen..

[65] Moyer H.R., Kinney R.C., Singh K.A., Williams J.K., Schwartz Z., Boyan B.D. (2010). Alginate microencapsulation technology for the percutaneous delivery of adipose-derived stem cells. Ann. Plast. Surg..

[66] Dar A., Shachar M., Leor J., Cohen S. (2002). Optimization of cardiac cell seeding and distribution in 3D porous alginate scaffolds. Biotechnol. Bioeng..

[67] Vasvani S., Kulkarni P., Rawtani D. (2019). Hyaluronic acid: A review on its biology, aspects of drug delivery, route of administrations and a special emphasis on its approved marketed products and recent clinical studies. Int. J. Biol. Macromol..

[68] Alberts B., Bray D., Hopin K., Johnson A., Lewis J., Raff M., Roberts K., Walter P. (2004). Tissues and cancer. Essential Cell Biology.

[69] Highley C.B., Prestwich G.D., Burdick J.A. (2016). Recent advances in hyaluronic acid hydrogels for biomedical applications. Curr. Opin. Biotechnol..

[70] Kobayashi H., Watanabe R., Choyke P.L. (2014). Improving conventional enhanced permeability and retention (EPR) effects; what is the appropriate target?. Theranostics.

[71] Qasim M., Arunkumar P., Powell H.M., Khan M. (2019). Current research trends and challenges in tissue engineering for mending broken hearts. Life Sci..

[72] Nguyen T.P., Nguyen Q.V., Nguyen V.H., Le T.H., Huynh V.Q., Vo D.V., Trinh Q.T., Kim S.Y., Le Q.V. (2019). Silk Fibroin-Based Biomaterials for Biomedical Applications: A Review. Polymers.

[73] Qi Y., Wang H., Wei K., Yang Y., Zheng R.-Y., Kim I.S., Zhang K.-Q. (2017). A Review of Structure Construction of Silk Fibroin Biomaterials from Single Structures to Multi-Level Structures. Int. J. Mol. Sci..

[74] Kim S.H., Yeon Y.K., Lee J.M., Chao J.R., Lee Y.J., Seo Y.B., Sultan M.T., Lee O.J., Lee J.S., Yoon S.I. (2018). Precisely printable and biocompatible silk fibroin bioink for digital light processing 3D printing. Nat. Commun..

[75] Guarino V., Gentile G., Sorrentino L., Ambrosio L. (2002). Polycaprolactone: Synthesis, properties, and applications. Encycl. Polym. Sci. Technol..

[76] Dai N.-T., Williamson M.R., Khammo N., Adams E.F., Coombes A.G. (2004). Composite cell support membranes based on collagen and polycaprolactone for tissue engineering of skin. Biomaterials.

[77] Fujihara K., Kotaki M., Ramakrishna S. (2005). Guided bone regeneration membrane made of polycaprolactone/calcium carbonate composite nano-fibers. Biomaterials.

[78] Kweon H., Yoo M.K., Park I.K., Kim T.H., Lee H.C., Lee H.S., Oh J.S., Akaike T., Cho C.S. (2003). A novel degradable polycaprolactone networks for tissue engineering. Biomaterials.

[79] Pok S., Benavides O.M., Hallal P., Jacot J.G. (2014). Use of myocardial matrix in a chitosan-based full-thickness heart patch. Tissue Eng. Part A.

[80] Allen C., Han J., Yu Y., Maysinger D., Eisenberg A. (2000). Polycaprolactone–b-poly (ethylene oxide) copolymer micelles as a delivery vehicle for dihydrotestosterone. J. Control. Release.

[81] Lopes M.S., Jardini A., Maciel Filho R. (2012). Poly (lactic acid) production for tissue engineering applications. Procedia Eng..

[82] Cui W., Cheng L., Hu C., Li H., Zhang Y., Chang J. (2013). Electrospun poly (L-lactide) fiber with ginsenoside rg3 for inhibiting scar hyperplasia of skin. PLoS ONE.

[83] Hart C.E., Loewen-Rodriguez A., Lessem J. (2012). Dermagraft: Use in the treatment of chronic wounds. Adv. Wound Care.

[84] Zhu G., Lou W. (2014). Regeneration of facial nerve defects with xenogeneic acellular nerve grafts in a rat model. Head Neck.

[85] Kim H.W., Lee H.H., Knowles J. (2006). Electrospinning biomedical nanocomposite fibers of hydroxyapatite/poly (lactic acid) for bone regeneration. J. Biomed. Mater. Res. Part A.

[86] Gentile P., Chiono V., Carmagnola I., Hatton P.V. (2014). An overview of poly (lactic-co-glycolic) acid (PLGA)-based biomaterials for bone tissue engineering. Int. J. Mol. Sci..

[87] Nassif L., El Sabban M. (2011). Mesenchymal stem cells in combination with scaffolds for bone tissue engineering. Materials.

[88] Ergenç T.I., Kizilel S. (2011). Recent advances in the modeling of PEG hydrogel membranes for biomedical applications. Biomedical Engineering, Trends in Materials Science.

[89] Soppimath K.S., Aminabhavi T.M., Dave A.M., Kumbar S.G., Rudzinski W. (2002). Stimulus-responsive “smart” hydrogels as novel drug delivery systems. Drug Dev. Ind. Pharm..

[90] Lutolf M., Hubbell J. (2005). Synthetic biomaterials as instructive extracellular microenvironments for morphogenesis in tissue engineering. Nat. Biotechnol..

[91] Tessmar J.K., Göpferich A.M. (2007). Customized PEG-derived copolymers for tissue-engineering applications. Macromol. Biosci..

[92] Lin C.-C., Anseth K.S. (2009). PEG hydrogels for the controlled release of biomolecules in regenerative medicine. Pharm. Res..

[93] Harris J.M. (2013). Poly (Ethylene Glycol) Chemistry: Biotechnical and Biomedical Applications.

[94] Nguyen L.H., Kudva A.K., Guckert N.L., Linse K.D., Roy K. (2011). Unique biomaterial compositions direct bone marrow stem cells into specific chondrocytic phenotypes corresponding to the various zones of articular cartilage. Biomaterials.

[95] Mahoney M.J., Anseth K.S. (2006). Three-dimensional growth and function of neural tissue in degradable polyethylene glycol hydrogels. Biomaterials.

[96] Rafat M., Li F., Fagerholm P., Lagali N.S., Watsky M.A., Munger R., Matsuura T., Griffith M. (2008). PEG-stabilized carbodiimide crosslinked collagen–chitosan hydrogels for corneal tissue engineering. Biomaterials.

[97] Salehi-Nik N., Rad M.R., Nazeman P., Khojasteh A. (2017). Polymers for oral and dental tissue engineering. Biomater. Oral Dent. Tissue Eng..

[98] Allen A.B., Priddy L.B., Li M.T., Guldberg R.E. (2015). Functional augmentation of naturally-derived materials for tissue regeneration. Ann. Biomed. Eng..

[99] Rnjak-Kovacina J., Wise S.G., Li Z., Maitz P.K., Young C.J., Wang Y., Weiss A.S. (2012). Electrospun synthetic human elastin:collagen composite scaffolds for dermal tissue engineering. Acta Biomater..

[100] Bharadwaz A., Jayasuriya A.C. (2020). Recent trends in the application of widely used natural and synthetic polymer nanocomposites in bone tissue regeneration. Mater. Sci. Eng. C.

[101] Malikmammadov E., Tanir T.E., Kiziltay A., Hasirci V., Hasirci N. (2017). PCL and PCL-based materials in biomedical applications. J. Biomater. Sci. Polym. Ed..

[102] Andorko J.I., Jewell C.M. (2017). Designing biomaterials with immunomodulatory properties for tissue engineering and regenerative medicine. Bioeng. Transl. Med..

[103] Lam M.T., Wu J.C. (2012). Biomaterial applications in cardiovascular tissue repair and regeneration. Expert Rev. Cardiovasc. Therapy.

[104] Kohn D.H., Sarmadi M., Helman J.I., Krebsbach P.H. (2002). Effects of pH on human bone marrow stromal cellsin vitro: Implications for tissue engineering of bone. J. Biomed. Mater. Res..

[105] Chan B.P., Leong K.W. (2008). Scaffolding in tissue engineering: General approaches and tissue-specific considerations. Eur. Spine J..

[106] Shah A., Brugnano J., Sun S., Vase A., Orwin E. (2008). The Development of a Tissue-Engineered Cornea: Biomaterials and Culture Methods. Pediatr. Res..

[107] Griffith M. (1999). Functional Human Corneal Equivalents Constructed from Cell Lines. Science.

[108] Karageorgiou V., Kaplan D. (2005). Porosity of 3D biomaterial scaffolds and osteogenesis. Biomaterials.

[109] Akay G., Birch M.A., Bokhari M.A. (2004). Microcellular polyHIPE polymer supports osteoblast growth and bone formation in vitro. Biomaterials.

[110] O’Brien F.J. (2011). Biomaterials & scaffolds for tissue engineering. Mater. Today.

[111] Prasadh S., Wong R.C.W. (2018). Unraveling the mechanical strength of biomaterials used as a bone scaffold in oral and maxillofacial defects. Oral Sci. Int..

[112] Zhao H., Liang W. (2017). A novel comby scaffold with improved mechanical strength for bone tissue engineering. Mater. Lett..

[113] Sartuqui J., D’Elía N., Gravina A.N., Messina P.V. (2015). Analyzing the hydrodynamic and crowding evolution of aqueous hydroxyapatite-gelatin networks: Digging deeper into bone scaffold design variables. Biopolymers.

[114] Stevens M.M., George J.H. (2005). Exploring and engineering the cell surface interface. Science.

[115] Hong H., Stegemann J.P. (2008). 2D and 3D collagen and fibrin biopolymers promote specific ECM and integrin gene expression by vascular smooth muscle cells. J. Biomater. Sci. Polym. Ed..

[116] Geckil H., Xu F., Zhang X., Moon S., Demirci U. (2010). Engineering hydrogels as extracellular matrix mimics. Nanomedicine.

[117] Tsang V.L., Bhatia S.N. (2004). Three-dimensional tissue fabrication. Adv. Drug Deliv. Rev..

[118] Ruso J., Messina P. (2016). Biopolymers in Regenerative Medicine: Overview, Current Advances and Future Trends.

[119] Langer R., Tirrell D.A. (2004). Designing materials for biology and medicine. Nature.

[120] Mani G., Feldman D.M., Patel D., Agrawal C.M. (2007). Coronary stents: A materials perspective. Biomaterials.

[121] Melek L.N. (2015). Tissue engineering in oral and maxillofacial reconstruction. Tanta Dent. J..

[122] Niaounakis M. (2013). Biopolymers: Reuse, recycling, and disposal. William Andrew.

[123] Patel D. (2011). Regenerative medicine using nanotechnology: A review. Int. J. Pharm. Biol. Arch..

[124] Rathenow J., Ban A., Kunstmann J., Mayer B., Asgari S. (2008). Biocompatible Coated Medical Implants.

[125] Ratner B.D., Hoffman A.S., Schoen F.J., Lemons J.E. (2004). Biomaterials Science: An Introduction to Materials in Medicine.

[126] Stevens M.M. (2008). Biomaterials for bone tissue engineering. Mater. Today.

[127] Lim G.T., Valente S.A., Hart-Spicer C.R., Evancho-Chapman M.M., Puskas J.E., Horne W.I., Schmidt S.P. (2013). New biomaterial as a promising alternative to silicone breast implants. J. Mech. Behav. Biomed. Mater..

[128] Terzic A., Nelson T.J. (2010). Regenerative medicine: Advancing healthcare 2020. J. Am. Coll. Cardiol..

[129] Tseng D., Donahue W., Parsons B.A. (2000). Polymer Coated Stent.

[130] White S.R., Sottos N.R., Geubelle P.H., Moore J.S., Kessler M.R., Sriram S.R., Brown E.N., Viswanathan S. (2001). Autonomic healing of polymer composites. Nature.

[131] Yu L., Ding J. (2008). Injectable hydrogels as unique biomedical materials. Chem. Soc. Rev..

[132] Zhou H., Lawrence J.G., Bhaduri S.B. (2012). Fabrication aspects of PLA-CaP/PLGA-CaP composites for orthopedic applications: A review. Acta Biomater..

[133] Zilla P., Bezuidenhout D., Human P. (2007). Prosthetic vascular grafts: Wrong models, wrong questions and no healing. Biomaterials.

[134] Ryan C.M., Schoenfeld D.A., Malloy M., Schulz J.T.I., Sheridan R.L., Tompkins R.G. (2002). Use of integra(R) artificial skin is associated with decreased length of stay for severely injured adult burn survivors. J. Burn Care Res..

[135] Tan H.P., Chu C.R., Payne K.A., Marra K.G. (2009). Injectable in situ forming biodegradable chitosan-hyaluronic acid based hydrogels for cartilage tissue engineering. Biomaterials.

[136] Johnson P.C. (1996). De novo cartilage generation using calcium alginate-chondrocyte constructs. Plast. Reconstr. Surg..

[137] Cruz D.M.G., Ivirico J.L.E., Gomes M.M., Ribelles J.L.G., Sanchez M.S., Reis R.L., Mano J.F. (2008). Chitosan microparticles as injectable scaffolds for tissue engineering. J. Tissue Eng. Regen. Med..

[138] Ahlmann E., Patzakis M., Roidis N., Shepherd L., Holtom P. (2002). Comparison of anterior and posterior iliac crest bone graft in terms of harvest-site morbidity and functional outcomes. J. Bone Jt. Surg..

[139] St John T.A., Vaccaro A.R., Sah A.P., Schaefer M., Berta S.C., Albert T., Hilibrand A. (2003). Physical and monetary costs associated with autogenous bone graft harvesting. Am. J. Orthop..

[140] Younger E.M., Chapman M.W. (1989). Morbidity at bone graft donor sites. J. Orthop. Trauma.

[141] Finkemeier C.G. (2002). Bone-grafting and bone-graft substitutes. J. Bone Jt. Surg..

[142] Giannoudis P.V., Dinopoulos H., Tsiridis E. (2005). Bone substitutes: An update. Injury.

[143] Komatsu D.E., Warden S.J. (2010). The control of fracture healing and its therapeutic targeting: Improving upon nature. J. Cell. Biochem..

[144] Chen J., Xu J., Wang A., Zheng M. (2009). Scaffolds for tendon and ligament repair: Review of the efficacy of commercial products. Expert Rev. Med Devices.

[145] Longo U.G., Lamberti A., Petrillo S., Maffulli N., Denaro V. (2012). Scaffolds in tendon tissue engineering. Stem Cells Int..

[146] Zhang X., Bogdanowicz D., Erisken C., Lee N.M., Lu H.H. (2012). Review Biomimetic scaffold design for functional and integrative tendon repair. J. Shoulder Elb. Surg..

[147] Liu Y., Ramanath H.S., Wang D.A. (2008). Tendon tissue engineering using scaffold enhancing strategies. Trends Biotechnol..

[148] Davidenko N., Schuster C.F., Bax D.V., Farndale R.W., Hamaia S., Best S.M., Cameron R.E. (2016). Evaluation of cell binding to collagen and gelatin: A study of the effect of 2D and 3D architecture and surface chemistry. J. Mater. Sci. Mater. Med..

[149] Yang J., Ding C., Tang L., Deng F., Yang Q., Wu H., Chen L., Ni Y., Huang L., Zhang M. (2020). Novel modification of collagen: Realizing desired water solubility and thermostability in a conflict-free way. ACS Omega.

[150] Lee D.W., Lim C., Israelachvili J.N., Hwang D.S. (2013). Strong adhesion and cohesion of chitosan in aqueous solutions. Langmuir.

[151] Mahapatro A., Singh D. (2011). Biodegradable Nanoparticles are Excellent Vehicle for Site Directed in-vivo Delivery of Drugs and Vaccines. J. Nanobiotechnol..

[152] Yang J.-S., Xie Y.-J., He W. (2011). Research progress on chemical modification of alginate: A review. Carbohydr. Polym..

[153] Zhu Y., Gao C., Liu X., Shen J. (2002). Surface modification of polycaprolactone membrane via aminolysis and biomacromolecule immobilization for promoting cytocompatibility of human endothelial cells. Biomacromolecules.

[154] Kosik-Kozioł A., Graham E., Jaroszewicz J., Chlanda A., Kumar P.S., Ivanovski S., Swieszkowski W., Vaquette C. (2018). Surface modification of 3D printed polycaprolactone constructs via a solvent treatment: Impact on physical and osteogenic properties. ACS Biomater. Sci. Eng..

[155] Rasal R.M., Janorkar A.V., Hirt D.E. (2010). Poly(lactic acid) modifications. Prog. Polym. Sci..

[156] Tasaka F., Miyazaki H., Ohya Y., Ouchi T. (1999). Synthesis of comb-type biodegradable polylactide through depsipeptide–lactide copolymer containing serine residues. Macromolecules.

[157] Lanchero R., Godoy-Silva R., Guerero C.A. Degradation kinetics of PLGA and PLGA conjugated with alendronate nanoparticles. Proceedings of the 2016 AIChE Annual Meeting, Nanoscale Science and Engineering Forum, Nanotechnology for Biotechnology and Pharmaceuticals.

[158] Cenni E., Micieli D., Fotia C., Salerno M., Granchi D., Avnet S., Sarpietro M.G., Castelli F., Baldini N. (2009). A novel biomaterial for osteotropic drug nanocarriers: Synthesis and biocompatibility evaluation of a PLGA–ALE conjugate Rosario Pignatello. Nanomedicine.

[159] Han Y., Yuan Z., Zhang P., Jiang S. (2018). Zwitterlation mitigates protein bioactivity loss in vitro over PEGylation. Chem. Sci..

[160] Bailon P., Won C.Y. (2009). PEG-modified biopharmaceuticals. Expert Opin. Drug Deliv..

